# Supplementary material for: The genome sequence of the Bloxworth Snout, Hypena obsitalis (Hübner, 1813) (Lepidoptera: Erebidae)
Source: Wellcome Open Res. 2026 Sep 14;11:657. [Version 1] doi: 10.12688/wellcomeopenres.27811.1 (PMC13752443; doi:10.12688/wellcomeopenres.27811.1)
Supplement: Peer review history

# Reviewer report: Reviewer response for version 1

**DOI:** 10.21956/wellcomeopenres.30604.r175900

**Published:** 2026-11-10

**Authors:** Maurijn van der Zee

**Affiliations:** 1 Leiden University, South Holland, The Netherlands

This is a high-quality genome, and all relevant data have been reported (metadata on the sample, methods, and metrics including genome size, BUSCO completeness, chromosomes, number of protein coding genes etc.). It will be a useful resource for future studies.

 I only have two questions for the authors: 

 -I wonder if it is possible to say something about the base accuracy (QV)?

 -How does sex determination work in this species? Is it that the females are the heterogametic sex (WZ)? As a male has been sequenced, am I then correct that the W chromosome has not been sequenced? Can the authors comment on this?

Are the datasets clearly presented in a usable and accessible format, and the assembly and annotation available in an appropriate subject-specific repository?

Yes

Are sufficient details of the sequencing and extraction, software used, and materials provided to allow replication by others?

Yes

Are the rationale for sequencing the genome and the species significance clearly described?

Yes

Are the protocols appropriate and is the work technically sound?

Yes

Reviewer Expertise:

evo-devo, insects, comparative genomics

I confirm that I have read this submission and believe that I have an appropriate level of expertise to confirm that it is of an acceptable scientific standard.

# Reviewer report: Reviewer response for version 1

**Published:** 2026-09-14

**Authors:** Automated Benchmarking

**Affiliations:** 1 Wellcome Sanger Institute, Hinxton, UK

As this article is a Genome Note, Automated Benchmarking has been used to determine whether the reported assembly passes three defined checks established by the Earth BioGenome Project: the raw and assembled data are publicly available, the Earth BioGenome Project three-part metric is met, and the BUSCO score passes the stated threshold. For Hypena obsitalis (ilHypObsi1), all three checks passed. Data available: pass. ENA study PRJEB82332, raw-read accessions ERR13946514 and ERR13947543, and assembly accessions GCA_965112525.1 and GCA_965112605.1 are public. Earth BioGenome Project three-part metric: pass. Haplotype 1 is reported as 6.C.Q65, meeting the 6.C.Q40 reference standard. BUSCO: pass. Haplotype 1, which is proposed as the reference assembly for this species, has 98.5% complete BUSCOs (97.9% single-copy and 0.6% duplicated). BUSCO software version: 5.5.0; lineage dataset: lepidoptera_odb10. It meets the target of greater than 90% complete and single-copy BUSCOs. This decision uses values reported in the Genome Note and public accession records.

Reviewer Expertise:

NA

I confirm that I have read this submission and believe that I have an appropriate level of expertise to confirm that it is of an acceptable scientific standard.
